# Clinical predictive factors for vaginal delivery following induction of labour among pregnant women in Jordan

**DOI:** 10.1186/s12884-021-04151-3

**Published:** 2021-10-07

**Authors:** Rawan A. Obeidat, Mahmoud Almaaitah, Abeer Ben-Sadon, Dina Istaiti, Hasan Rawashdeh, Shereen Hamadneh, Hanan Hammouri, Adel Bataineh

**Affiliations:** 1 Department of Obstetrics and Gynecology, Faculty of Medicine, Jordan University of Science and Technology, King Abdullah University Hospital, P. O. Box: 3030, Irbid, 22110 Jordan; 2 Department of Maternal and Child Health, Al Al-Bayt University, Mafraq, Jordan; 3 Department of Mathematics and Statistics, Jordan University of Science and Technology, Irbid, Jordan; 4 Department of Anesthesia, Jordan University of Science and Technology, Irbid, Jordan

**Keywords:** Induction of labour, Parity, Prostaglandin E2, Bishop score, Cesarean section

## Abstract

**Background:**

Induction of labour (IOL) is an important and common clinical procedure in obstetrics. In the current study, we evaluate predictors of vaginal delivery in both nulliparous and multiparous women in north Jordan who were induced with vaginal prostaglandins.

**Method:**

A prospective study was conducted on 530 pregnant women at King Abdullah University Hospital (KAUH) in north Jordan. All pregnant mothers with singleton live fetuses, who had induction of labour (IOL) between July 2017 and June 2019, were included in the study. Mode of delivery, whether vaginal or caesarean, was the primary outcome. Several maternal and fetal variables were investigated. The safety and benefit of repeated dosage of vaginal prostaglandin E2 (PGE2) tablets, neonatal outcomes and factors that affect duration of labour were also evaluated.

Pearson χ2 test was used to investigate the significance of association between categorical variables, while student’s t-test and ANOVA were applied to examine the mean differences between categorical and numerical variables. Linear regression analysis was utilized to study the relation between two continuous variables. A multivariate regression analysis was then performed. Significance level was considered at alpha less than 0.05.

**Results:**

Nulliparous women (*N* = 254) had significantly higher cesarean delivery rate (58.7% vs. 17.8%, *p* < 0.001) and longer duration of labour (16.1 ± 0.74 h vs. 11.0 ± 0.43 h, *p* < 0.001) than multiparous women (*N* = 276). In nulliparous women, the rate of vaginal delivery was significantly higher in women with higher Bishop score; the mean Bishop score was 3.47 ± 0.12 in nulliparous women who had vaginal delivery vs. 3.06 ± 0.10 in women who had cesarean delivery (Adjusted odds ratio (AOR) = 1.2, 95% CI: 1.03–1.28, *p* = 0.03). In multiparous women, the rate of vaginal delivery was significantly higher in women with higher Bishop scores and lower in women with higher body mass index (BMI). The mean Bishop score was 3.97 ± 0.07 in multiparous women who had vaginal delivery vs. 3.56 ± 0.16 in women who had cesarean delivery (AOR = 1.5, 95% CI: 1.1–2.1, *p* = 0.01). The mean BMI was 30.24 ± 0.28 kg/m^2^ in multiparous women who had vaginal delivery vs. 32.36 ± 0.73 kg/m^2^ in women who had cesarean delivery (AOR = 0.89, 95% CI: 0.84–0.96, *p* = 0.005). 27% of nulliparous women who received more than two PGE2 tablets and 50% of multiparous women who received more than two PGE2 tablets had vaginal delivery with no significant increase in neonatal morbidity.

**Conclusion:**

Parity and cervical status are the main predictors of successful labour induction. Further studies are required to investigate the benefit of the use of additional doses of vaginal PGE2 above the recommended dose for IOL.

## Background

Induction of labour (IOL) is an important and common clinical procedure in obstetrics. The frequency of induction is estimated to be 15–25% [1]. IOL is usually indicated when the benefits of delivery to the mother or fetus outweigh the potential risks of continuing the pregnancy, however, the benefit of induction is not always clear. Post-term pregnancy is the most common indication for induction. There are mechanical and pharmacological methods used to induce labour. Prostaglandins have been demonstrated to be highly effective in achieving cervical ripening and vaginal delivery [2].

Induction of labour has been associated with an increased risk of cesarean delivery [3–6]. Nulliparous women are particularly at increased risk [7, 8]. Thus, there has been considerable interest in predicting delivery outcomes in women assessed prior to IOL. Several factors have been reported to be associated with a higher chance of successful induction including a favourable cervix, multiparity, ruptured membranes, lower body mass index (BMI), taller height, younger age, increased gestational age and lower estimated fetal weight [7, 9–11].

The purpose of our study was to investigate the relevant factors for vaginal delivery among Jordanian women with prostaglandin-induced labour. The primary outcome was the rate of vaginal delivery. The secondary outcomes were time from induction to delivery and the safety of the use of additional doses of vaginal prostaglandin E2 (PGE2) tablets above the recommended dose for IOL.

## Material and methods

### Design

A prospective study was conducted to evaluate predictors of vaginal delivery in both nulliparous and multiparous women in north Jordan who were induced with prostaglandins. The safety and benefit of repeated dosage of vaginal PGE2 tablets (Dinoprostone), neonatal outcomes and factors that affect duration of labour were also evaluated.

### Data collection

Our study has been carried out at King Abdullah University Hospital (KAUH) in north Jordan. The study method and protocol were approved in May 2017 by the Institutional Review Board of the hospital (Approval no. 465/2017). The study was performed in accordance with the Code of Ethics in the Declaration of Helsinki. All pregnant women with singleton live fetuses, who had induction of labour (IOL) between July 2017 and June 2019, were included in the study. None of our patients had a previous cesarean delivery.

Written informed consents were obtained from pregnant women. Data was collected by registrars in the labour ward. The data collected include maternal age, parity, blood group, haemoglobin level, maternal weight and height, interval between last pregnancy and current pregnancy, previous miscarriages, previous preterm deliveries, previous successful induction of labour (IOL), gestational age of the fetus at the time of induction, indications for induction, Bishop score and status of the membranes (intact or ruptured) at the time of induction, membranes sweeping in the last 7 days before induction, method of induction, the dosage of PGE2 tablets received, delivery mode, duration of labour, indications of cesarean section, neonatal weight and gender, Apgar score at 1 and 5 min and neonatal admission to neonatal intensive care unit (NICU).

530 pregnant women were included in the study and all of them had a Bishop score of 6 or less. 461 women (87%) were induced by vaginal administration of PGE2 tablets, 67 women (12.6%) were induced by vaginal administration of PGE2 controlled-release pessary (Propess), and 2 women (0.4%) were induced initially with Propess that were removed because of hyperstimulation and then PGE2 tablets were used later. All our patients were admitted to labour ward during the process of induction. Patients were induced with vaginal administration of Propess once for 24 h or two vaginal PGE2 tablets (3 mg) at 6-h intervals. Some multiparous women received half a tablet (1.5 mg). If the cervix remained unfavourable after the second dose of PGE2 tablets, some consultants performed cesarean sections while others counselled their patients regarding the administration of a third dose of PGE2; if they agreed to continue IOL then a third dose of PGE2 tablets was given; otherwise, cesarean sections were performed. Amniotomy ± oxytocin augmentation were performed in cases with unsatisfactory progress of labour. In most patients, amniotomy was performed when cervical dilation was ≥2 cm. Oxytocin was started following amniotomy and at least 4–6 h after the last dose of prostaglandins. Oxytocin infusion was initiated at 1–2 milliunits/minute (mU/min) and was increased by 1–2 mU/min every 30 min until effective uterine contractions (*3 contractions in 10 min lasting for 40 s*) were achieved or 32 mU/min was reached. Continuous electronic fetal heart rate monitoring was performed during active labour.

### Statistical analysis

Statistical analysis was performed using IBM SPSS Statistics Software (v.26), 2019. Data were presented as frequency distributions for categorical variables and mean ± standard error of the mean for continuous variables. Pearson χ2 test was used to investigate the significance of association between categorical variables, while student’s t-test and ANOVA were applied to examine the mean differences between categorical and numerical variables. Simple linear regression analysis was utilized to study the relation between two continuous variables. After that, multivariate analysis using binary logistic regression was performed to evaluate each variable as an independent predictor of delivery mode (vaginal vs. cesarean). Adjusted Odds ratio (OR) and their corresponding 95% confidence intervals (CI) were calculated. Multivariate analysis using a linear regression model was also performed to determine the most significant predictors for delivery duration. All variables with *p* ≤ 0.25 on univariate analysis were included in the multivariate analysis. Significance level was considered at alpha less than 0.05. The Kolmogorov–Smirnov test was used to test for normality of continuous variables.

The analysis was performed for the whole group (*N* = 530) and then further analysis was performed for the subgroups: multiparous (*N* = 276) women and nulliparous (*N* = 254) women.

## Results

During the study period, 530 pregnant women were admitted to the labour ward for induction of labour. Postdate was the most common indication (23.4%) followed by rupture of membranes (premature rupture of membranes (PROM) / preterm premature rupture of membranes (PPROM) (21.1%). The mean age of women was 29.2 years. The mean gestational age of fetuses was 39 weeks with a range of 33.3–42.4 weeks. 332 women (62.6%) had vaginal delivery with a mean duration of labour of 12.7 h and 198 women (37.4%) had cesarean section (Table 1). The mean weight of neonates was 3.1 kg with a range of 1.6–4.2 kg, 78 neonates (14.7%) were admitted to NICU, the main reason for admission was observation or treatment of infection in neonates delivered to mothers with a history of confirmed or suspected rupture of membranes more than 18 h. The mean Apgar score at 1 min and 5 min were 7.9 and 9.1 respectively. The main indication for cesarean section was fetal distress (46%); other indications were failure to progress (19.7%), failed IOL (18.2%) and maternal request (14.6%).

**Table 1:** Outcomes of induction of labour (IOL) for all women (*N* = 530)

Outcome	Frequency (%)
	Mean ± SE
Mode of delivery	
• Vaginal delivery	332 (62.6)
• Cesarean section	198 (37.4)
Duration of labor (hours)	12.7 **±** 0.39
Apgar score at 1 min	7.9 **±** 0.04
Apgar score at 5 min	9.1 **±** 0.02
Admission to NICU	78 (14.7)
Indication for NICU admission	
• Observation / Antibiotics (ruptured membranes / sepsis)	63 (11.9)
• Respiratory distress (RDS / TTN)	7 (1.3)
• Low Apgar for observation	3 (0.6)
• Meconium aspiration	1 (0.2)
• Low weight for observation	2 (0.4)
• Shoulder dystocia	1 (0.2)
• Suspected TEF	1 (0.2)
Indication of cesarean sections	
• Fetal distress	91 (17.2)
• Failure to progress	39 (7.4)
• Failed IOL^a^	36 (6.8)
• Maternal request	29 (5.5)
• Others	3 (0.6)

^a^ Failed IOL: defined as the inability to achieve the active phase of labour, NICU: neonatal intensive care unit; RDS: respiratory distress syndrome; TTN: transient tachypnea of newborn; TEF: tracheoesophageal fistula; SE: standard error of the mean

Of those 530 women, 254 women (47.9%) were nulliparous and 276 women (52.1%) were multiparous. Compared with multiparous women, nulliparous women had significantly higher cesarean delivery rate (58.7% vs. 17.8%, *p* < 0.001) and longer duration of labour (16.1 ± 0.74 h vs. 11.0 ± 0.43 h, *p* < 0.001). However, there was no significant difference in the frequency of neonatal admission to NICU and Apgar score at 1 and 5 min. Neonatal weight (3.2 ± 0.03 kg vs. 3.0 ± 0.03 kg, *p* = 0.002) was significantly higher in the multiparous group.

1. **Mode of delivery**

(A) **Nulliparous (N = 254)**

In nulliparous women, the rate of vaginal delivery was significantly higher in women with higher Bishop score (OR = 1.2, 95% CI: 1.03–1.28, *p* = 0.03). The mean Bishop score was 3.47 ± 0.12 in nulliparous women who had vaginal delivery vs. 3.06 ± 0.10 in women who had cesarean delivery. Other factors were not shown to be significantly associated with mode of delivery in nulliparous women (Tables 2 and 3).

**Table 2:** Maternal and fetal variables and their association with mode of delivery in induction of labour in multiparous and nulliparous women

Variables	Multiparous (*N* = 276)				Nulliparous (*N* = 254)			
	Frequency	VD (%)	CS (%)	*p*-value ^a^	Frequency	VD (%)	CS (%)	*p*-value ^a^
Previous miscarriage	116	93 (80.2)	23 (19.8)	0.43	40	15 (37.5)	25 (62.5)	0.59
Previous preterm delivery	14	13 (92.9)	1 (7.1)	0.29	NA			
Previous IOL	125	103 (82.4)	22 (17.6)	0.83	NA			
Rupture (confirmed) of the membranes at time of induction								
• All cases with ruptured membranes	18	17 (94.4)	1 (5.6)	0.16	30	15 (50)	15 (50)	0.31
• PROM	14	14 (100)	0 (0)	0.08	19	11 (57.9)	8 (42.1)	0.22
• PPROM	4	3 (75)	1 (25)	0.77	11	4 (36.4)	7 (63.6)	0.22
Membranes sweeping in the last 7 days	67	55 (82.1)	12 (17.9)	0.87	41	20 (48.8)	21 (51.2)	0.26
Method of induction used								
• Propess	45	36 (80)	9 (20)	0.10	22	11 (50)	11 (50)	0.33
• PGE2 tablet	230	191 (83)	39 (17)		231	93 (40.3)	138 (59.7)	
• Propess + PGE2 table	1	0 (0)	1 (100)		1	1 (100)	0 (0)	
Blood group								
• A	101	84 (83.2)	17 (16.8)	0.51	99	35 (35.4)	64 (64.6)	0.33
• AB	18	17 (94.4)	1 (5.6)		14	9 (64.3)	5 (35.7)	
• B	47	38 (80.9)	9 (19.1)		43	20 (46.5)	23 (53.5)	
• O	110	88 (80.0)	22 (20.0)		98	41 (41.8)	57 (58.2)	
Indication for IOL								
• Postdate	64	54 (84.4)	10 (15.6)	0.32	60	25 (41.7)	35 (58.3)	0.51^c^
• Rupture of membranes (confirmed & suspected)	52	46 (88.5)	6 (11.5)		60	27 (45.0)	33 (55.0)	
• Reduced liquor	35	30 (85.7)	5 (14.3)		42	15 (35.7)	27 (64.3)	
• Reduced fetal movement	37	27 (73.0)	10 (27.0)		35	13 (37.1)	22 (62.9)	
• SGA / IUGR	13	8 (61.5)	5 (38.5)		17	8 (47.1)	9 (52.9)	
• Hypertension	14	12 (85.7)	2 (14.3)		14	8 (57.1)	6 (42.9)	
• Thrombophilia	13	11 (84.6)	2 (15.4)		5	2 (40.0)	3 (60.0)	
• GDM / DM	19	14 (73.7)	5 (26.3)		12	4 (33.3)	8 (66.7)	
• Intrahepatic cholestasis of pregnancy	6	6 (100)	0 (0)		6	0 (0)	6 (100)	
• Antepartum haemorrhage	3	3 (100)	0 (0)		1	1 (100)	0 (0)	
• Others (e.g., maternal request, unstable lie, etc.)	20	16 (80)	4 (20)		2	1 (50)	1 (50)	
Neonate gender								
• Male	131	110 (84)	21 (16)	0.55	115	46 (40.0)	69 (60.0)	0.59
• Female	145	117 (80.7)	28 (19.3)		139	58 (42.4)	80 (57.6)	
**Variables**	**Mean ± SE**							
	**VD**	**CS**		***p*- value**^**b**^	**VD**	**CS**		***p*- value**^**b**^
Age (years)	32 ± 0.33	33.3 ± 0.85		0.15	25.53 ± 0.38	26.23 ± 0.32		0.17
Bishop score	3.97 ± 0.07	3.59 ± 0.16		0.033	3.47 ± 0.12	3.06 ± 0.10		0.01
Interval between last pregnancy and current pregnancy (months)	40.07 ± 1.77	50.57 ± 5.72		0.09	NA			
Hemoglobin at time of IOL (g/dl)	11.06 ± 0.08	11.05 ± 0.17		0.93	11.5 ± 0.127	11.7 ± 0.08		0.19
Mother height (cm)	162.40 ± 0.35	162.86 ± 0.76		0.67	161.80 ± 0.57	161.9 ± 0.52		0.87
Mother weight at time of IOL (kg)	79.7 ± 0.75	86.04 ± 2.24		0.01	77.85 ± 1.3	78.7 ± 1.1		0.61
Mother BMI at time of IOL (kg/m^2^)	30.2 ± 0.28	32.36 ± 0.73		0.009	29.68 ± 0.45	30.02 ± 0.38		0.56
Gestational age of the fetus (weeks)	40.48 ± 1.6	38.67 ± 0.25		0.60	38.94 ± 0.16	38.56 ± 0.15		0.09
Neonate weight (kg)	3.17 ± 0.03	3.16 ± 0.08		0.96	3.07 ± 0.04	3.02 ± 0.04		0.35

VD: vaginal delivery; CS: cesarean section; IOL: induction of labour; PGE2: prostaglandin E2; SGA: small for gestational age; IUGR: intrauterine growth restriction; GDM: gestational diabetes; DM: diabetes mellitus; SE: standard error of the mean.

^a^ Two-sided *p-*value based on univariate analysis – Chi-square and t-test

^b^ Two-sided *p-*value based on univariate analysis – simple linear regression

^c^ *p*-value = 0.036 for nulliparous women with intrahepatic cholestasis of pregnancy

**Table 3:** Multivariate analysis of factors affecting delivery mode in induction of labour

Factors	OR^a^	95% CI	*p*-value^b^
**Nulliparous**			
Age (years)	0.95	0.86–1.01	0.12
Hemoglobin at time of IOL (g/dl)	0.82	0.65–1.01	0.07
Gestational age of the fetus (weeks)	1.12	0.95–1.31	0.18
Bishop Score	1.23	1.03–1.28	0.03
Status of the membrane at the time of induction	1.1	0.69–1.6	0.27
Indication for induction - Intrahepatic cholestasis of pregnancy	0.1	0.67–1.49	0.79
**Multiparous**			
Maternal BMI at time of IOL (kg/m^2^)	0.89	0.84–0.96	0.005
Bishop Score	1.5	1.1–2.1	0.01
Interval between last pregnancy and current pregnancy (months)	0.99	0.98–1.0	0.19
Method of induction used	1.1	0.46–2.8	0.97
Status of the membrane at time of induction	0.14	0.15–1.2	0.72

CI: confidence interval; IOL: induction of labour, BMI: body mass index.

^a^ OR: odds ratio of vaginal delivery

^b^ Two-sided *p*-value based on binary logistic regression adjusted for all factors shown

(B) **Multiparous (** ***N*** **= 276)**

In multiparous women, the rate of vaginal delivery was significantly higher in women with higher Bishop score (OR = 1.5, 95% CI: 1.1–2.1, *p* = 0.01) and lower in women with higher BMI (OR = 0.89, 95% CI: 0.84–0.96, *p* = 0.005). The mean Bishop score and BMI were 3.97 ± 0.07 and 30.24 ± 0.28 kg/m^2^ respectively in multiparous women who had vaginal delivery vs. 3.56 ± 0.16 and 32.36 ± 0.73 kg/m^2^ in women who had cesarean delivery. No other variables were found to be significantly associated with mode of delivery in multiparous women (Tables 2 and 3).

2. **Number of PGE2 tablets used**

(A) **Nulliparous (** ***N*** **= 231)**

231 nulliparous women were induced by PGE2 tablets (Table 4). Of these, 37 (16%) received more than two PGE2 tablets. 27% of women who received more than two PGE2 tablets had vaginal delivery. Compared with women who received two or fewer PGE2 tablets; Apgar score at 1 min was significantly better (8.3 ± 0.13 vs. 7.8 ± 0.08, *p* = 0.007), there was no significant difference in Apgar score at 5 min (*p* = 0.18), however, there was a more frequent, though not significant, neonatal admission to NICU (24.3% vs. 17.5%, *p* = 0.33) mainly because of the higher percentage of prolonged (> 18 h) ruptured membranes in women where more than two PGE2 tablets were used.

(B) **Multiparous (*N*** **= 230)**

**Table 4:** Outcomes of induction of labour in both multiparous and nulliparous women (*N* = 461) and their relation to the number of prostaglandins E2 tablets used

Number of PGE2 tablets		Multiparous (N = 230)			Nulliparous (*N* = 231)		
		≤ 2 tablets	> 2 tablets	*p*-value ^a^	≤ 2 tablets	> 2 tablets	*p*-value ^a^
**Frequency (%)**							
**Women induced with PGE2 tablets**		208 (90.4)	22 (9.6)	NA	194 (84)	37 (16)	NA
**Mode of delivery**	**VD**	180 (86.5)	11 (50)	< 0.001	83 (42.8)	10 (27)	0.073
	**CS**	28 (13.5)	11 (50)		111 (57.2)	27 (73)	
**Admission to neonatal intensive care unit**		25 (12)	5 (22.7)	0.156	34 (17.5)	9 (24.3)	0.33
**Mean ± SE**							
**Apgar score at 1 min**		8.0 ± 0.05	8.0 ± 0.26	0.96	7.8 ± 0.08	8.3 ± 0.13	0.007
**Apgar score at 5 min**		9.1 ± 0.04	9.1 ± 0.11	0.88	9.0 ± 0.04	9.2 ± 0.09	0.18

^a^ Two-sided *p-*value based on univariate analysis – Chi-square and t-test

PGE2: prostaglandin E2; SE: standard error of the mean; VD: vaginal delivery; CS: cesarean section.

As shown in Tables 4, 230 multiparous women were induced by PGE2 tablets, 22 (9.6%) women received more than two PGE2 tablets. 50% of women who received more than two PGE2 tablets had vaginal deliveries. Compared with women who received two or fewer PGE2 tablets; there was no significant difference in Apgar score at 1 min (*p* = 0.96) and 5 min (*p* = 0.88), however, there was a more frequent, but not significant, neonatal admission to NICU (22.7% vs. 12%, *p* = 0.156) mainly because of the higher percentage of prolonged (> 18 h) ruptured membranes in women where more than two PGE2 tablets were used.

3. **Duration of labour**

(A) **Nulliparous (** ***N*** **= 105)**

105 nulliparous women had vaginal delivery, the majority of these delivered within 24 h; 31 women (29.5%) gave birth within 12 h, 62 women (59%) within 12–24 h and 12 (11.4%) women within 24–48 h from the beginning of induction.

**Fig. 1:**
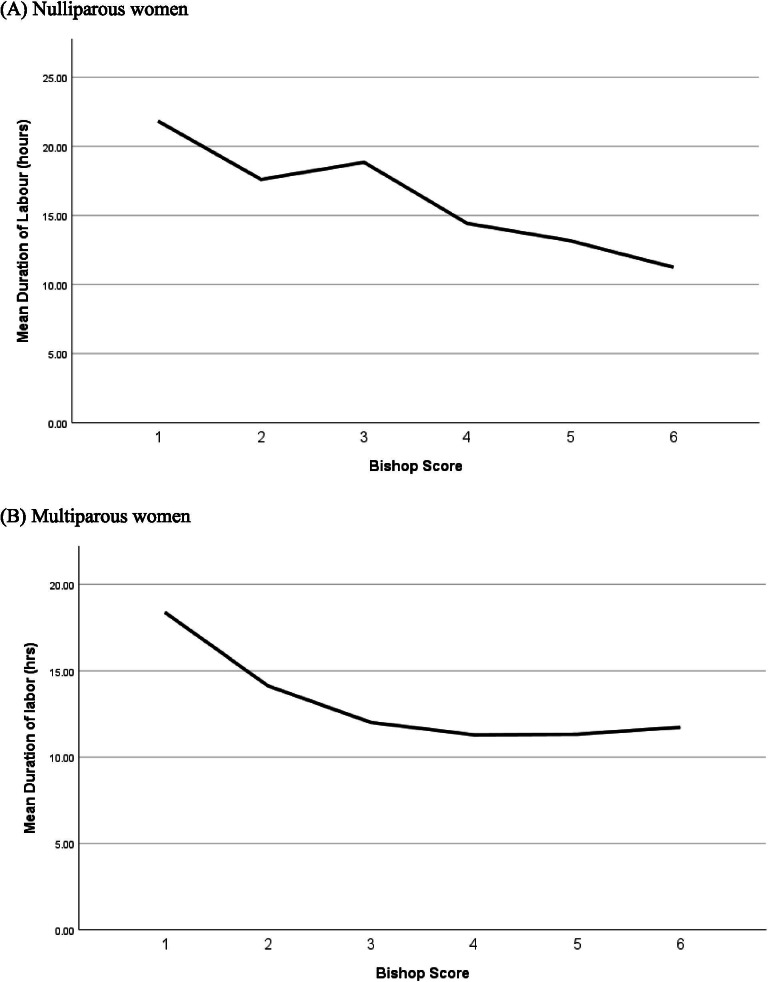
The relation between Bishop score and duration of labour in nulliparous and multiparous women

A higher Bishop score was significantly associated with a shorter duration of labour (*P* < 0.001) in nulliparous women (Fig. 1) (Table 5).

**Table 5:** Multivariate analysis of factors affecting delivery duration in induction of labour

Predictor	Unstandardized Coefficients (β)	Standardized Coefficients (Beta)	Standard Error	*p*-value ^a^	95% CI
**Nulliparous**					
Bishop score	−2.1	−0.337	0.58	< 0.001	−3.25 – −1.03
**Multiparous**					
Bishop score	−0.85	−0.133	0.421	0.045	−1.70 – −0.030
Previous preterm delivery	3.65	0.132	1.81	0.049	0.07–7.23

^a^ Two-sided *p-*value based on multivariate analysis – linear regression

CI: confidence interval.

(B) **Multiparous (** ***N*** **= 227)**

227 multiparous women had vaginal delivery, the majority of these delivered within 12 h; 166 women (73.1%) gave birth within 12 h, 50 women (22%) within 12–24 h and 11 women (4.8%) within 24–48 h from the beginning of induction.

Induction to vaginal delivery time interval was significantly shorter in women with higher Bishop score (*p* = 0.045) and longer in women who had previous preterm deliveries (*p* = 0.049) (Fig. 1) (Table 5).

In both nulliparous and multiparous women, there was no significant association between induction to delivery interval and: previous miscarriages, the status of the membranes at the time of induction, membrane sweeping in the last 7 days prior to IOL, type of prostaglandins used, maternal blood group, indications of induction, neonate gender and weight, maternal age, maternal height and weight, maternal haemoglobin level, and gestational age of the fetus.

## Discussion

Induction of labour is a widely used clinical procedure in obstetrics. This study was performed to evaluate maternal and fetal characteristics that independently predict a successful induction of labour, defined as vaginal delivery.

Induction of labour has been associated with an increased risk of cesarean delivery [3–5, 8] However, this association has been challenged in recent years [7, 12, 13]. In term and post-term pregnancies, the risk of cesarean delivery was shown to be lower among women who were induced than among those managed expectantly [13]. Also, compared with expectant management, IOL among low-risk nulliparous women at ≥39 weeks was shown to be significantly associated with a lower risk of cesarean delivery, reduced risk of maternal peripartum infection and hypertensive disorders of pregnancy and lower perinatal adverse outcomes, including respiratory morbidity, intensive care unit admission, and mortality [14–16]. A vaginal delivery rate of 62.6% was reported in our study and is lower than the rates observed in most of the studies in the literature [2]. However, some studies showed low vaginal birth rates, for example, Osmundson et al. reported a 56.9% vaginal delivery rate among induced nulliparous women [16]; similarly, a vaginal delivery rate of 68.4% was reported in a prospective study in China [17]. Our low vaginal birth rate could be because our institution (KAUH) is a tertiary referral centre receiving high-risk pregnancies. Moreover, 14.6% of cesarean deliveries were on maternal request and possibly further patients counselling by health care providers regarding IOL should be encouraged in our institution.

Parity is the most important variable implicated in a successful IOL. Studies are consistent in demonstrating higher success rates of induction in multiparous compared to nulliparous women [5, 18–26]. Our study agrees with existing literature that ‘multiparity’ is linked with the success of IOL and showed a vaginal delivery of 82.2% among multiparous women vs. 41.3% among nulliparous women.

Higher Bishop score in our study was a protective factor for vaginal delivery in both nulliparous and multiparous group. Bishop score is one of the best available tools for assessing cervical status [5, 11]. Most studies reported an inverse relationship between Bishop score and failure of labour induction, with low scores being associated with a higher cesarean delivery rate [1, 3, 5, 7, 18, 19, 22, 24, 25, 27] and longer induction to vaginal delivery time interval [23]. However, other studies reported that Bishop score was a poor predictor of the outcome of labour in women scheduled for induction [23, 28, 29].

Body mass index is an important parameter in clinical practice. In our study, lower maternal weight and BMI were predictive of vaginal delivery in multiparous women but not in nulliparous women. In the literature, higher BMI was reported to be associated with a longer duration of labour [19, 20, 28] and a higher cesarean delivery rate [1, 3, 5–7, 11, 20, 24, 27, 28]. On the contrary, other studies showed that weight and BMI did not influence the likelihood of successful induction [18, 23, 27].

Maternal height has been identified by some investigators as predictive of vaginal delivery; taller women were reported to have a better chance of success for IOL [7, 9–11, 19]. However other studies showed no effect of maternal height on the likelihood of successful induction [23]. Our study reported no association between maternal height and the mode of delivery in both nulliparous and multiparous women.

Younger maternal age was found to be predictive of the success of IOL in many studies [3, 5, 7, 11, 18, 19, 25] but not in the other studies [1, 26, 27]. In our study, elder maternal age was significantly associated with a higher vaginal delivery rate in the whole sample, but this significance disappeared when adjusted for parity. Analysis of subgroups showed that maternal age is not an independent predictor of IOL in both nulliparous and multiparous group.

Characteristics of the fetus or neonate may also influence the success of induction. Successful induction was reported to be associated with increased gestational age [7, 13, 19, 23] and lower birth weight [5, 7, 11, 18, 19, 21, 23, 24]. However, in our study and other studies fetal gestational age [1, 26] and neonatal weight [26–28] were not predictors of success of induction.

Rupture of membranes has been reported to be associated with a higher chance of successful induction. Though, in other studies membranes status was not associated with successful labour induction [11]. In our study, neither all cases of ruptured membranes (*confirmed by clinical exam + suspected cases based on history but not confirmed by exam*) nor confirmed cases of ruptured membranes were associated with the success of induction. Sub-analysis of term and preterm pregnancies showed similar results (Table 2).

In the current study, there was no difference in the rate of vaginal delivery between controlled-release pessary (Propess) and vaginal PGE2 tablet. Similarly, A Cochrane review conducted by Thomas et al. reported no evidence of differences in vaginal delivery rates with 24 h and cesarean section rates between PGE2 controlled-release pessary and either vaginal PGE2 tablet or gel [2]. The oral administration of PGE2 tablets is not authorized in our institution as studies showed it was no more effective in achieving vaginal delivery than other routes, had more frequent gastrointestinal adverse effects and there was no clear evidence favouring its use regarding the safety of women and fetuses [30–32]. Alike, misoprostol use for labour induction is not licensed in our institution, however, the oral use of low dose misoprostol has been recommended by many studies. In a Cochrane review conducted by Alfirevic et, a total of 3240 women were randomised to oral misoprostol or vaginal dinoprostone in 10 trials, and the results showed no statistically significant differences between the two groups in any of the following outcomes: achieving vaginal birth within 24 h, cesarean birth rates and uterine hyperstimulation rates [32]. Moreover, other studies favour the use of oral misoprostol and they revealed that although oral misoprostol was associated with a longer induction to vaginal delivery interval than vaginal PGE2 [33, 34], it was safer, since it resulted in fewer cesarean sections [33–35] and less uterine hyperstimulation with fetal heart changes [34, 35].

In our study, there was no significant association between history of membrane stripping in the last week and the rate of vaginal delivery in IOL. We recognized that part of women who had sweeping had spontaneous labour and they did not need IOL. Stripping of the membranes is associated with a reduced frequency of post-term pregnancies [36–38] and should not be discouraged.

There is no full agreement on the ideal dosage of PGE2 that should be used in IOL. The National Institute for Health and Care Excellence (NICE) recommended regimen for IOL by PGE2 tablets is a maximum of two doses 6 h apart [30]. Our study showed that, reassuringly, the use of additional doses of PGE2 tablets above the recommended dose for induction of labour was not associated with increased neonatal morbidity and nearly a third of nulliparous women and a half of multiparous women achieved vaginal delivery. Similarly, two recent retrospective studies showed that repeated doses of vaginal PGE2 for IOL above the current recommendations was associated with a higher vaginal delivery rate without compromising neonatal wellbeing for both nulliparous and multiparous women [39, 40].

## Study limitation

Our study group includes women with both complicated and uncomplicated pregnancies, term and preterm pregnancies, intact and ruptured membranes, different methods of induction (PGE2 tablets and Propess) and different indications for IOL. Having a heterogeneous study group may be considered a drawback, however, it allowed us to evaluate many factors in their association with IOL and also we consider our results as reliable due to the use of suitable statistical analysis. Another limitation is that maternal morbidity was not fully assessed in our study.

## Conclusion

Despite the large number of studies performed, there are still many controversies regarding factors implicated in a successful IOL. Parity and cervical status are the main predictors of successful labour induction. Improving the prediction of induction failure is currently a major challenge in obstetrics and is mandatory to improve IOL management and outcome. IOL for inappropriate indication should be discouraged especially before 39 weeks and in nulliparous women with an unfavourable cervix.

Further studies are required to investigate the benefit of the use of additional doses of vaginal PGE2 above the recommended dose for IOL.
